# Low Shear Stress Induced HMGB1 Translocation and Release via PECAM-1/PARP-1 Pathway to Induce Inflammation Response

**DOI:** 10.1371/journal.pone.0120586

**Published:** 2015-03-20

**Authors:** Wei-dong Qin, Shao-hua Mi, Chen Li, Gui-xia Wang, Jian-ning Zhang, Hao Wang, Fan Zhang, Yang Ma, Da-wei Wu, Mingxiang Zhang

**Affiliations:** 1 The Department of Critical Care Unit, Qilu Hospital of Shandong University, Jinan, Shandong, China; 2 The Department of Cardiology, Yu Huang Ding Hospital, Yantai, Shandong, China,; 3 The Department of Radiology, Linyi People’s Hospital, Linyi, Shandong, China; 4 The Key Laboratory of Cardiovascular Remodeling and Function Research, Chinese Ministry of Education and Chinese Ministry of Public Health, Qilu Hospital of Shandong University, Jinan, Shandong, China; University of Padua, ITALY

## Abstract

Low shear stress (LSS) plays a critical role in the site predilection of atherosclerosis through activation of cellular mechanosensors, such as platelet endothelial cell adhesion molecule 1 (PECAM-1). Poly(ADP-ribose) polymerase 1 (PARP-1) is a nuclear enzyme that regulates the expression of various inflammatory cytokines. The nuclear enzyme high mobility group box 1 (HMGB1) can induce inflammation response by binding to toll-like receptor 4 (TLR4). In the present study, we aimed to investigate the role and mechanism of HMGB1 in LSS induced inflammation in human umbilical vein endothelial cells (HUVECs). HUVECs were stimulated by undisturbed shear stress (USS, 1 Pa) and LSS (0.4 Pa) in our experiments. Gene expression was inhibited by small interfering RNA (siRNA). ICAM-1 expression was regulated by LSS in a time dependent manner. LSS can induce HMGB1 translocation from nucleus to cytoplasm and release. Compared with the USS, LSS could increase the protein expression of PECAM-1 and PARP-1 as well as the secretion of TNF-α and IL-1β. LSS induced the translocation of HMGB1 from nucleus to cytoplasm. Inhibition of HGMB1 reduced LSS-induced inflammatory response. Inhibition of PARP-1 suppressed inflammatory response through inhibiting TLR4 expression and HMGB1 translocation. PECAM-1 inhibition reduced LSS-induced ICAM-1 expression, TNF-α and IL-1β secretion, and monocytes adhesion. LSS can induce inflammatory response via PECAM-1/PARP-1/HMGB1 pathway. PARP-1 plays a fundamental role in HMGB1 translocation and TLR4 expression. Inhibition of PARP-1 may shed light on the treatment of HMGB1 involved inflammation during atherosclerosis.

## Introduction

Atherosclerosis is a chronic inflammatory disease in the large and medium arteries. It is often associated with various risk factors, such as age, sex, and cigarette. Inflammatory cytokines play important roles in the development and progression of atherosclerosis.[1] Cellular adhesion molecules, such as intercellular adhesion molecule 1 (ICAM-1), contribute to the inflammatory response and endothelial dysfunction.[2] They can mediate the margination, adhesion and transendothelial migration of circulating monocytes from the blood stream to the vessel wall.[3] They can also recruit and activate monocytes to release matrix metalloproteinases (MMP), promote plaque rupture and initiate acute coronary syndromes.

The chronic inflammatory response localizes at specific sites of the vascular tree.[4] This site predilection is thought to be caused by hemodynamic parameters, especially the wall shear stress (WSS). WSS is a frictional force exerted parallel to the vessel wall.[5] It is directly proportional to the velocity of blood flow, and inversely proportional to the radius of the arterial. The value of physiological WSS ranges from 0.5 Pa to 1.2 Pa, while values below 0.5 Pa and above 1.2 Pa are considered to be low shear stress (LSS) and high shear stress (HSS), respectively.[6] WSS can be sensed by numerous mechanosensors on the luminal surface of endothelial cells.[7] Platelet endothelial cell adhesion molecule-1 (PECAM-1) has recently been considered to be an important kind of mechanosensors.[8] LSS can promote the transport of pro-atherogenic substances between the lumen and the vessel wall to induce atherosclerosis.[9] For many years, LSS has been considered to be a prediction of plaque formation during atherosclerosis.

Poly(ADP-ribose) polymerase 1 (PARP-1), the most abundant isoform of the PARP enzyme family, is a highly conserved DNA-binding nuclear enzyme.[10] Once activated by DNA strand breaks, PARP-1 catalyzes NAD into nicotinamide and ADP-ribose to form long branches of ADP-ribose polymers on itself and other nuclear chromatin-associated proteins.[11] Excessive activation of this enzyme results in the intracellular depletion of NAD and ATP, thus leading to cellular energy crisis, mitochondrial dysfunction and cell death.[12] PARP-1 has been shown to be associated with circulatory shock, heart failure, ischemia reperfusion injury, hypertension, and diabetes.[13,14,15,16]

High mobility group box 1 (HMGB1), a member of high mobility group nuclear proteins, is constitutively expressed in the nucleus of eukaryotic cells.[17] As a nuclear protein, HMGB1 plays intracellular and extracellular activities.[18] Inside the cells, HMGB1 can regulate the nucleosomal structure and stability, and the activity of transcription factors.[19,20] Outside the cells, HMGB1 is a potent endogenous alarm for innate immunity. The translocation of HMGB1 from the inside to the outside of the cells is a critical event in host defense and inflammation response.[21] HMGB1 can be either actively released by activated immune cells or passively released from damaged/necrotic cells.[22,23] There are several important receptors in HMGB1 signaling, including toll-like receptor (TLR) 2, TLR4 and the receptor for advanced glycation end products (RAGE).[24,25] HMGB1 has been implicated in the pathogenesis of autoimmune disorders, such as systemic lupus erythematosus, autoimmune diabetes, and arthritis.[26,27,28] However, its role in LSS induced inflammation has not been fully investigated.

In the present study, after human umbilical vein endothelial cells were stimulated by LSS (0.4 Pa), we investigated the role and mechanism of HMGB1 in LSS induced inflammation response.

## Materials and Methods

All experiments were performed in compliance with the Guide for the Care and Use of Laboratory Animals (NIH Publication No. 85–23, revised 1996) and were approved by the Ethics Committee of Shandong University.

### Cell culture, flow system and gene inhibition

Human umbilical vein endothelial cells (HUVECs, ATCC, USA) were cultured in endothelial cell medium (ECM, ScienCell, CA, USA) with 5% fetal bovine serum (FBS), 100 U/ml penicillin, and 100 μg/ml streptomycin at 37°C. Cells up to passage 4 were used and seeded onto gelatin-coated slides. A parallel-plate flow system was used to impose USS (1 Pa) and LSS (0.4 Pa).[29] The THP-1 monocytes were cultured in RPMI-1640 medium (GIBCO, CA, USA) containing L-glutamine (2 mmol/L) and 10% FBS as well as the same amounts of penicillin and streptomycin. To inhibit genes expression, cells were transfected with negative control of siRNA (si-NC) or siRNA (GenePharma, Shanghai, China) in Optimem Medium (Invitrogen, CA, USA) by using Lipofectamine 2000 (Invitrogen). Experiments were performed 24 hrs after transfection.

### RT-PCR

Cellular RNA was extracted by using Trizol reagent (Invitrogen). The cDNA was analyzed by real-time RT-PCR with SYBR Green Supermix (Bio-Rad Laboratories, CA, USA). Each sample was analyzed in triplicate, and expression was normalized to β-actin. The primers for ICAM-1 were as follows: forward, 5'-TTGGAAGCCTCATCCG-3', reverse, 5'-CAATGTTGCGAGACCC-3'; for HMGB1, forward, 5'- AAGCACCCAGATGCTTCAGT-3', reverse, 5'-TCCGCTTTTGCCATATCT-TC-3'; for PARP-1, forward, 5'-TTGAAAAAGCCCTAAAGGCTCA-3', reverse, 5'- CTACTCGGTCCAAGATCGCC-3', for PECAM-1, forward, 5'-GGAAGCCAACAGCCA-TTACGG-3', reverse, 5'-GAGCCTTCCGTTCTCTTGGTGA-3', and for β-actin, forward, 5'-TGGACATCCGCAAAGAC-3', reverse, 5'-GAAAGGGTGTAACGCAACTA-3'. Amplification, detection, and data analysis were performed by the use of the iCycler real-time PCR system (Bio-Rad Laboratories). The relative expression of genes was obtained by the 2-ΔΔCt calculation method.

### Western blot analysis

Total protein was extracted from HUVECs by use of RIPA Lysis Buffer (Beyotime, Nantong, China). Equal amount of protein was separated on 10% SDS-PAGE and transferred onto nitrocellulose membrane (Millipore, MA, USA). After being blocked with 5% non-fat milk for 2 hrs at room temperature, blots were washed with TBS-T for 3 times and incubated with the primary antibodies: rabbit monoclonal anti-β-actin (1:1000, Cell Signaling Technology, MA, USA) and anti-HMGB1 (1:1000, Abcam, MA, USA); rabbit polyclonal anti-PARP-1 (1:500, Sigma-Aldrich, MO, USA); goat anti-TLR4 (1:500; R&D, MN, USA); mouse monoclonal anti-ICAM-1 (1:500; Santa Cruz Biotechnology, CA, USA), and anti-PECAM-1 (1:500, Sigma) overnight at 4°C. After being washed with TBS-T, membranes were incubated with horseradish peroxidase-conjugated secondary antibody for 2 hrs at room temperature. Bands were visualized by enhanced chemiluminescence (Millipore) and analyzed by use of Image-Pro Plus 6.0. Each experiment was carried out for two times at least.

### Immunofluorescence

After stimulation, HUVECs were fixed in 4% paraformaldehyde and permeabilized in 0.1% Triton X-100/PBS for 5 min. After being blocked with BSA for 30 min, cells were incubated with the primary antibodies rabbit monoclonal anti-HMGB1 (1:1000, Abcam) and mouse monoclonal anti-ICAM-1 (1:500; Santa Cruz Biotechnology) overnight at 4°C. After being washed with TBS-T, cells were incubated with secondary antibodies (1:500; Jackson immunoresearch). A drop of Prolong Gold antifade reagent with DAPI (Vector Laboratories, CA, USA) was used to seal the coverslip. Images were acquired by laser scanning confocal microscopy (LSM 710; Carl Zeiss, Germany). Data were analyzed by use of Image-Pro Plus 6.0.

### ELISA

The cell culture supernatant was obtained by centrifugation at 1000 g for 10 min. The HMGB1 release, TNF-α and IL-1β secretion were determined by use of the ELISA kit (Uscn Life Science Inc., Wuhan, China) according to the manufacturer’s instructions. All operations were performed at room temperature. Mean absorbance for standards and samples was assessed in duplicate. The color reaction was detected by use of Varioskan Flash multifunction plate reader (Thermo Scientific, Rockford, USA).

### Monocyte adhesion assay

THP-1 monocytes (ATCC) were used in the adhesion assay as previously described.[30] Briefly, THP-1 cells (5×10^5^ cells/ml) were labeled with a fluorescent dye BCECF-AM (10 μM, Beyotime) in serum-free RPMI-1640 medium for 45 min at 37°C with frequent agitation. Following exposure to shear stress in the presence or absence of siRNA, the HUVECs monolayers were washed in RPMI-1640 medium before adding BCECF-AM loaded THP-1 cells. After incubation for 45 min at 37°C, unbound monocytes were removed by washing monolayers three times with PBS, followed by fixation and mounting with a glass coverslip. Bound monocytes were quantified by counting the cells under a fluorescent microscope.

### Statistical Analysis

Data are expressed as mean±SD. Analyses were performed on SPSS version 16.0 (SPSS Inc, Chicago, IL, USA) for Windows. Intergroup comparisons involved 2-tailed Student *t* test or one-way ANOVA followed by Tukey test (with equal variances assumed) or Dunnett T3 test (with equal variances not assumed). A *P*<0.05 was considered to indicate statistical significance.

## Results

### ICAM-1 expression was regulated by LSS in a time-dependent manner

**Fig 1:**
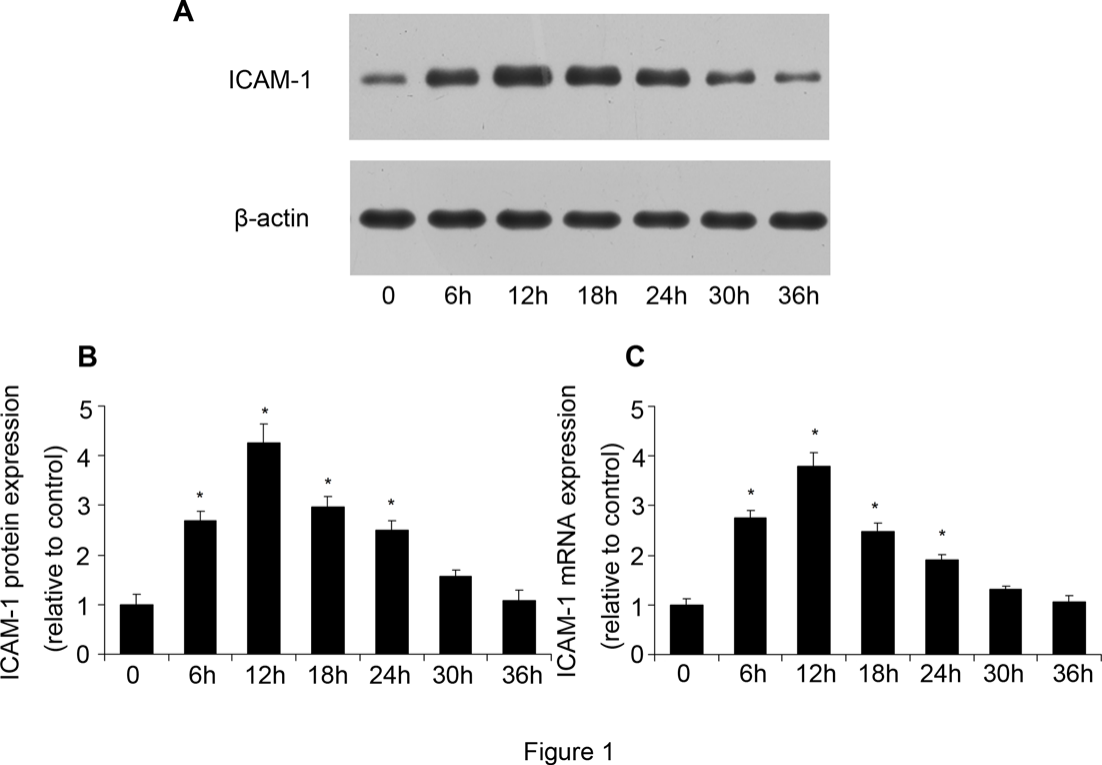
ICAM-1 expression was induced by low shear stress (LSS, 0.4 Pa). After HUVECs was stimulated by low shear stress for different times (0, 6, 12, 18, 24, 30, 36 h), ICAM-1 expression was determined by western blot analysis and RT-PCR. **(A, B)** Western blot analysis of ICAM-1 protein expression. **(C)** Quantification of ICAM-1 mRNA expression. Values are expressed as mean ± S.D. from three independent experiments. *P<0.05 vs. 0 h treatment.

After HUVECs were stimulated by LSS (0.4 Pa) for various times (0, 6, 12, 18, 24, 30, and 36 h), the protein and mRNA expression of ICAM-1 was determined by western blot analysis and RT-PCR. The protein expression of ICAM-1 began to increase at 6 h, while it peaked at 12 h and then decreased at 18 h (Fig. 1A and 1B). There was no significant difference in ICAM-1 expression between 0h and 36h stimulation. The mRNA expression of ICAM-1 showed a similar result (Fig. 1C). Therefore, HUVECs were stimulated by low shear stress for 12 h in the following experiments.

### LSS induced HMGB1 translocation and release

**Fig 2:**
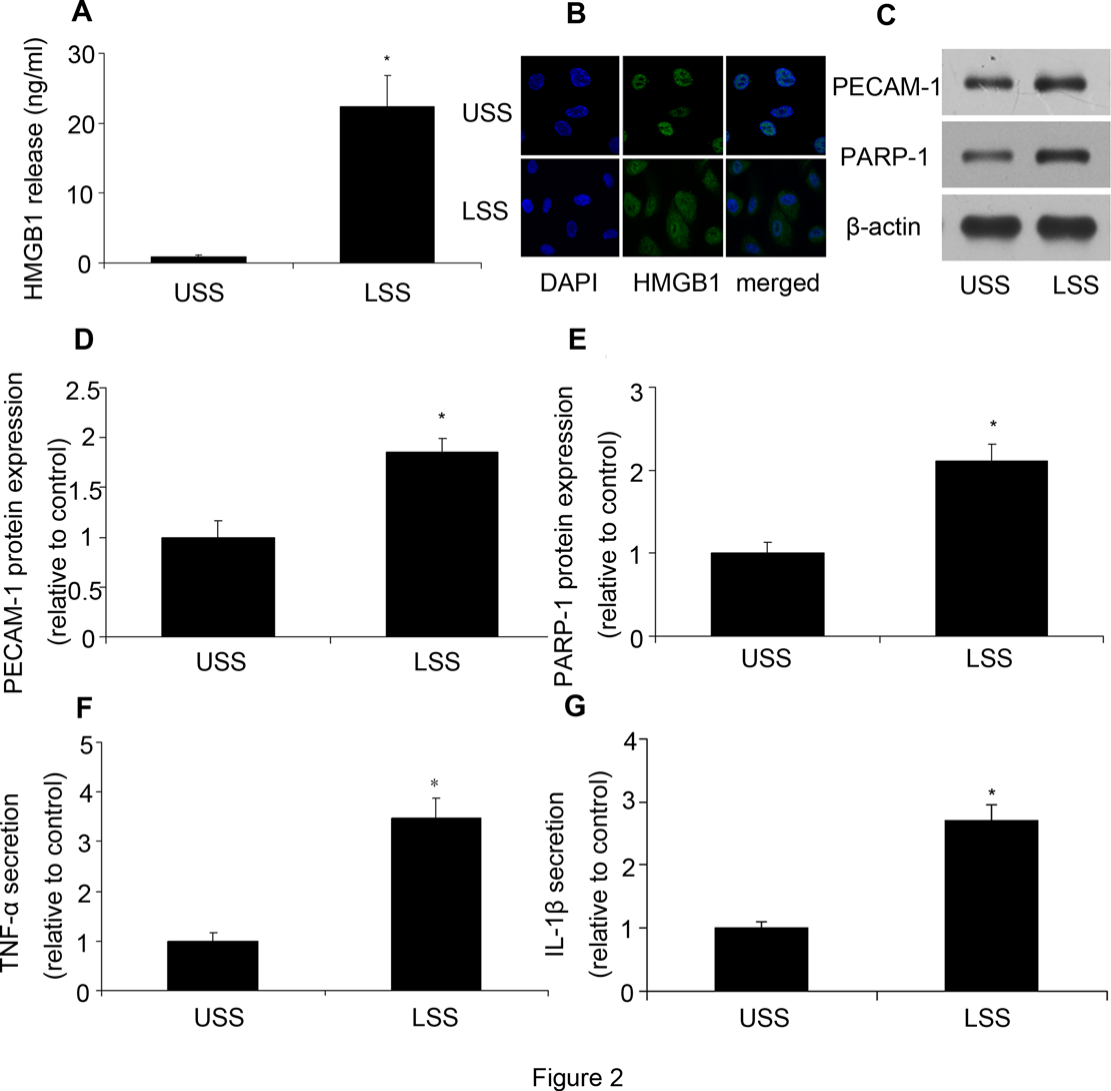
LSS induced HMGB1 translocation and release and upregulated protein expression and inflammatory cytokines secretion in HUVECs. After HUVECs were stimulated by LSS, HMGB1 content in supernatant and HMGB1 translocation were determined by ELISA and immunoflurescence, respectively. PECAM-1 and PARP-1 protein expression were determined by western blot analysis. TNF-α and IL-1β secretion were assessed by ELISA. **(A)** HMGB1 release was assessed by ELISA. **(B)** Immunofluorescence of HMGB1 protein expression. Nuclei were labelled with 4′,6-diamidino-2-phenylindole (DAPI) (blue); HMGB1 was stained with rabbit anti- HMGB1 primary antibody and Alexa 488-conjugated goat anti-rabbit second antibody (green). **(C, D, E)** Western blot analysis of PECAM-1 and PARP-1 protein expression. **(F)** TNF-α was assessed by ELISA. **(G)** IL-1β was assessed by ELISA. Values are expressed as mean ± S.D. *P<0.05 vs. USS.

As a nuclear protein, the translocation of HMGB1 from inside to the outside of cells plays a critical role in inflammation response. After HUVECs were exposed to LSS, HMGB1 expression in the culture supernatant and cells was assessed by ELISA and immunofluorescence, respectively. As we expected, there was almost no HMGB1 release from cells under USS, while LSS significantly increased HMGB1 concentration in the supernatant (Fig. 2A). Meanwhile, HMGB1 was mainly in nucleus under USS condition, but it translocated to the cytoplasm after LSS stimulation (Fig. 2B).

### LSS increased PECAM-1 and PARP-1 expression as well as TNF-α and IL-1β secretion

Then we detected the protein expression of PECAM-1 and PARP-1 as well as TNF-α and IL-1β secretion. Compared with the control, LSS could significantly upregulate PECAM-1 and PARP-1 protein expression (Fig. 2C-2E), while TNF-α and IL-1β secretion was also increased by LSS (Fig. 2F and 2G).

### HMGB1 inhibition reduced LSS-induced inflammatory response

**Fig 3:**
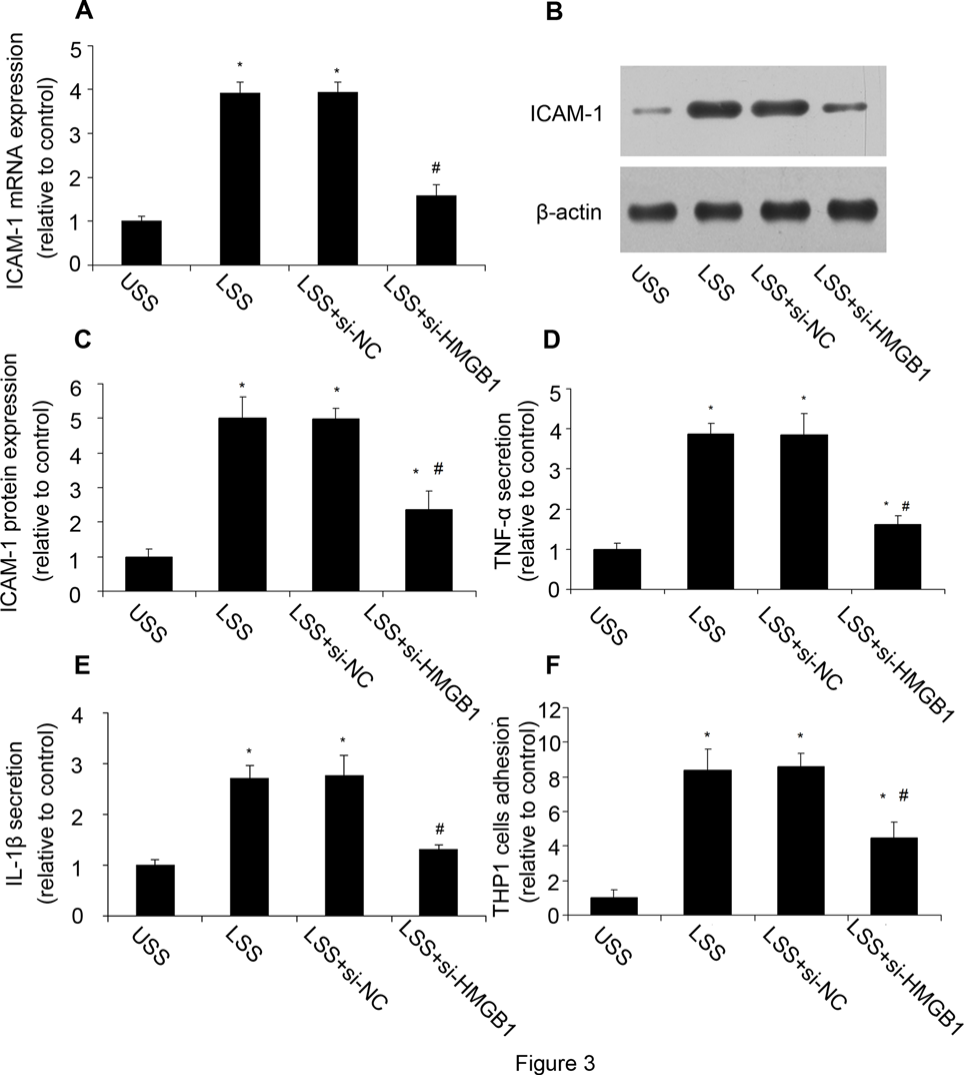
HMGB1 inhibition reduced LSS-induced inflammatory response. HMGB1 inhibition reduced LSS-induced inflammatory response, including ICAM-1 mRNA and protein expression, TNF-α and IL-1β secretion and monocytes adhesion. **(A, B, C)** The mRNA and protein expression of ICAM-1 were assessed by RT-PCR and western blot analysis. **(D)** TNF-α secretion was assessed by ELISA. **(E)** IL-1β secretion was assessed by ELISA. **(F)** THP-1 monocytes adhesion. Values are expressed as mean ± S.D. from three separate experiments. *P<0.05 vs. USS. #*P*<0.05 vs. LSS; si-HMGB1: HMGB1 siRNA; si-NC: negative control of HMGB1 siRNA.

To confirm our hypothesis that LSS induced inflammatory response via HMGB1, we used HMGB1 siRNA in the following experiments. Firstly we verified the validity of HMGB1 siRNA. The RT-PCR and western blot analysis showed that the mRNA and protein expression of HMGB1 was significantly reduced by siRNA (S1 Fig.). After HMGB1 was inhibited by siRNA, HUVECs were exposed to LSS. Compared with USS, LSS increased ICAM-1 expression, while HMGB1 inhibition decreased LSS-induced ICAM-1 expression (Fig. 3A-3C). Meanwhile, LSS could significantly increase the TNF-α and IL-1β secretion as well as the number of adhesional THP-1 cells, while HMGB1 inhibition reduced the inflammatory response (Fig. 3D-3F). The results suggested that HMGB1 played a critical role in LSS induced inflammation response.

### PARP-1 inhibition reduced LSS-induced inflammatory response

**Fig 4:**
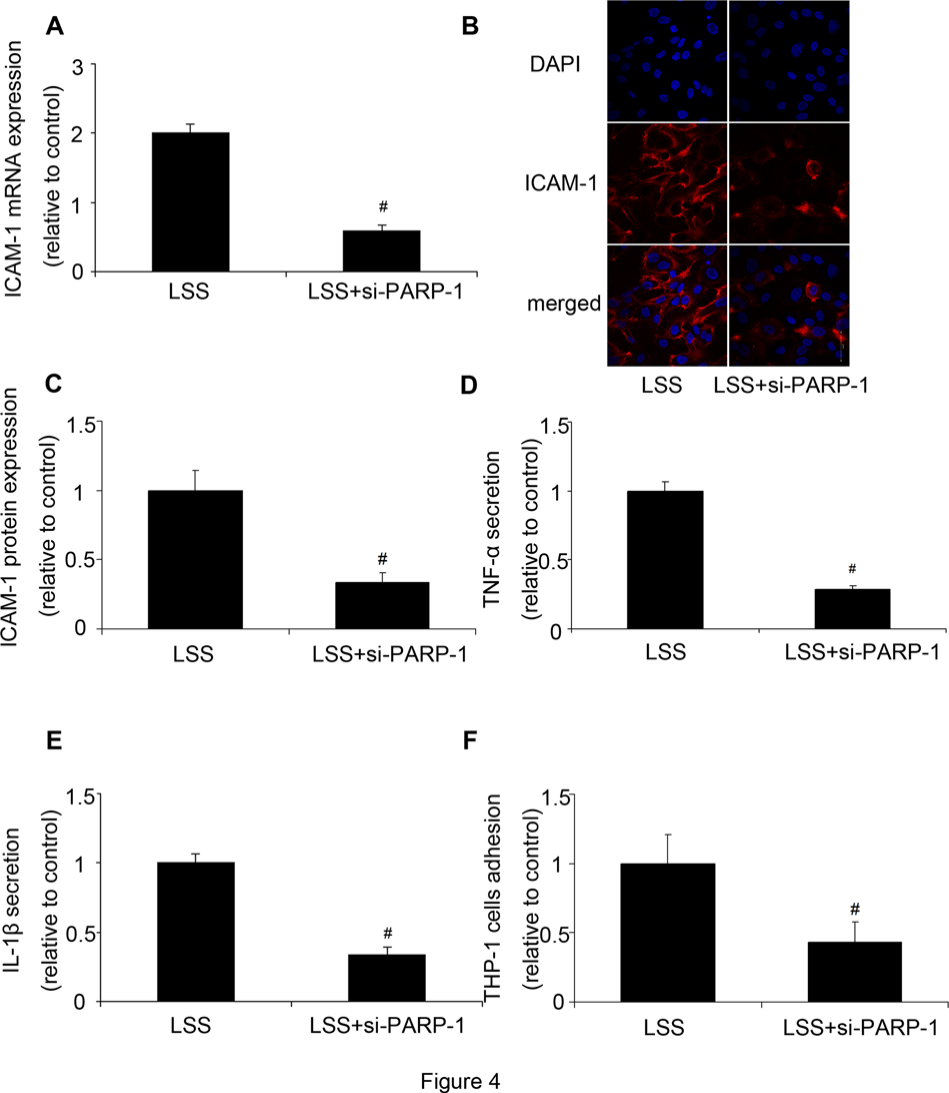
PARP-1 inhibition decreased LSS-induced inflammatory response. PARP-1 inhibition reduced LSS-induced inflammatory response. **(A, B, C)** The mRNA and protein expression of ICAM-1 were analyzed by RT-PCR and immunofluorescence. Nuclei were labelled with 4′,6-diamidino-2-phenylindole (DAPI) (blue); ICAM-1 was stained with mouse anti-ICAM-1 primary antibody and Alexa 555-conjugated goat anti-mouse second antibody (red). **(D)** TNF-α secretion was assessed by ELISA. **(E)** IL-1β secretion was assessed by ELISA. **(F)** THP-1 monocytes adhesion. Values are expressed as mean ± S.D. from two separate experiments. #*P*<0.05 vs. LSS; si-PARP-1: PARP-1 siRNA; si-NC: negative control of PARP-1 siRNA.

We then investigated the role of PARP-1 in LSS induced inflammation. Firstly we verified the validity of PARP-1 siRNA. Compared with the control, PARP-1 protein and mRNA expression were significantly reduced by PARP-1 siRNA, respectively (S2 Fig.). Subsequently, HUVECs were stimulated by LSS after PARP-1 was inhibited by siRNA. Compared with the LSS group, ICAM-1 expression was significantly reduced by PARP-1 siRNA (Fig. 4A-4C). Moreover, PARP-1 inhibition reduced LSS-induced TNF-α and IL-1β secretion as well as THP-1 monocytes adhesion (Fig. 4D-4F).

### PARP-1 inhibition reduced TLR4 expression and suppressed HMGB1 translocation and release

**Fig 5:**
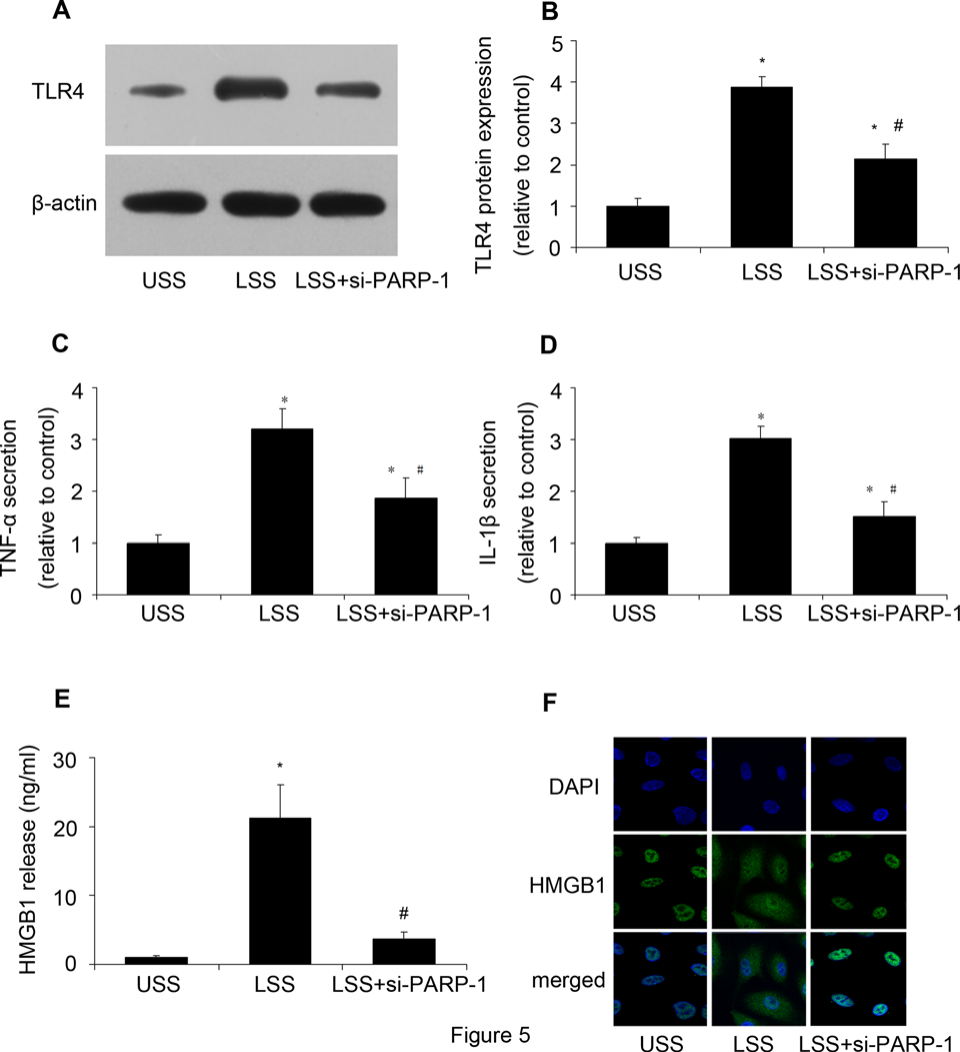
PARP-1 inhibition reduced TLR4 expression and suppressed HMGB1 translocation and release. After PARP-1 was inhibited by siRNA, HUVECs were stimulated by LSS, then TLR4 expression was determined by western blot analysis, TNF-α and IL-1β secretion were assessed by ELISA, HMGB1 release and HMGB1 translocation were assessed by ELISA and immunofluorescence, respectively. **(A, B)** The protein expression of TLR4 was assessed by western blot analysis. **(C)** TNF-α secretion was assessed by ELISA. **(D)** IL-1β secretion was assessed by ELISA. **(E)** HMGB1 release was determined by ELISA. **(F)** Immunofluorescence of HMGB1 protein expression. Nuclei were labelled with 4′,6-diamidino-2-phenylindole (DAPI) (blue); HMGB1 was stained with rabbit anti- HMGB1 primary antibody and Alexa 488-conjugated goat anti-rabbit second antibody (green). * *P*<0.05 vs. USS; # *P*<0.05 vs. LSS; si-PARP-1: PARP-1 siRNA.

Then we investigated the underlying mechanism. When released from cells, HMGB1 is a proinflammatory mediator by binding to receptors. TLR4 expression was determined by western blot analysis. Compared with the USS, LSS could increase TLR4 protein expression, while PARP-1 inhibition reduced it (Fig. 5A and 5B). Meanwhile, PARP-1 inhibition reduced LSS-upregulated TNF-α and IL-1β secretion (Fig. 5C and 5D). The results suggested that PARP-1 played a critical role in LSS induced TLR4 expression. Considering the important role of HMGB1 in inflammation, HMGB1 release and expression were determined by ELISA and immunofluorescence, respectively. As we expected, PARP-1 inhibition decreased LSS-induced HMGB1 release (Fig. 5E). Fig. 5F showed that PARP-1 inhibition suppressed the translocation of HMGB1 from nucleus to cytoplasm. These results suggested that PARP-1 inhibition could suppress HMGB1 translocation and release as well as TLR4 expression to alleviate LSS induced inflammation response.

### PECAM-1 inhibition reduced PARP-1 expression and inflammatory response

**Fig 6:**
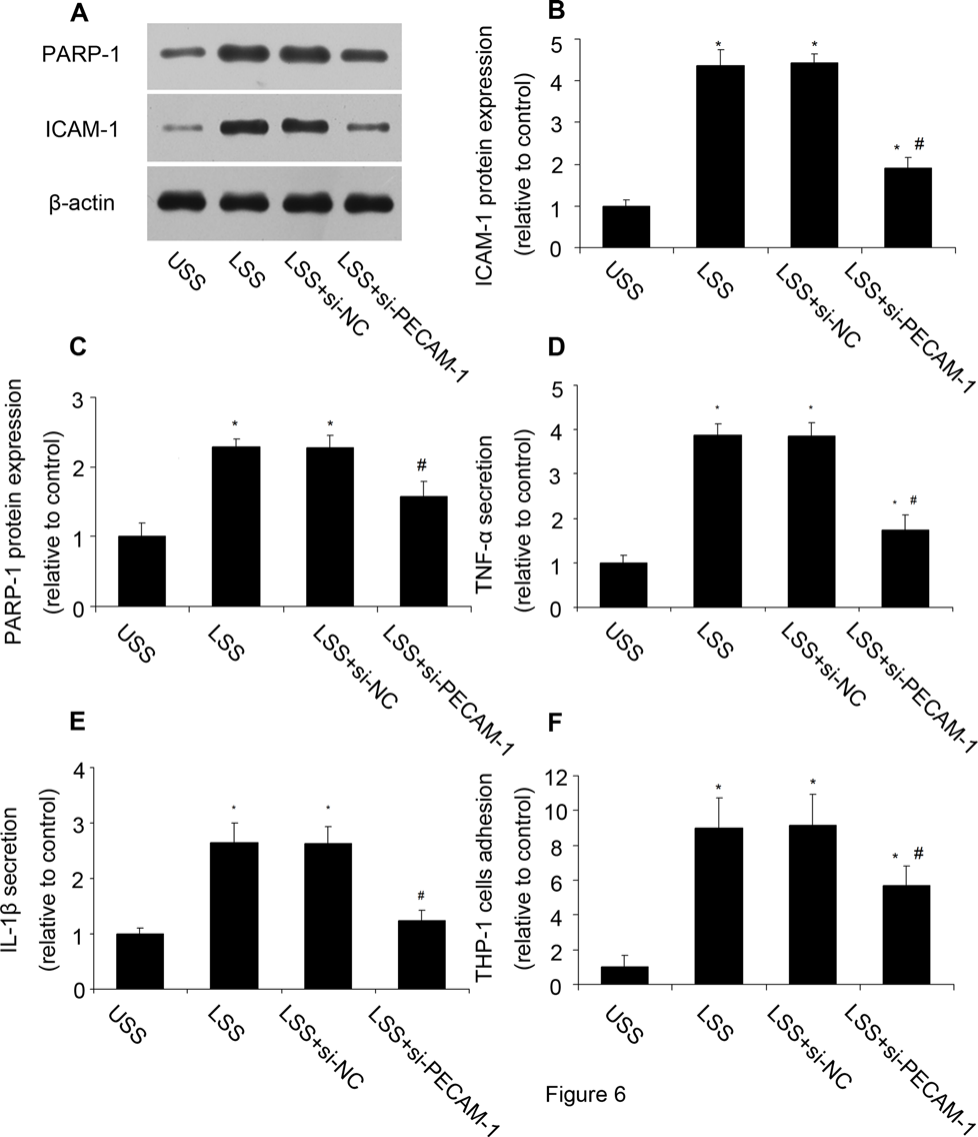
PECAM-1 inhibition decreased PARP-1 expression and inflammatory response. After PECAM-1 was inhibited by siRNA, PARP-1 expression and inflammatory response were determined. **(A, B, C)** The protein expression of PARP-1 and ICAM-1 was analyzed by western blot analysis. **(D)** TNF-α secretion was assessed by ELISA. **(E)** IL-1β secretion was assessed by ELISA. **(F)** THP-1 monocytes adhesion. Values are expressed as mean ± S.D. from three separate experiments. *P<0.05 vs. USS. #*P*<0.05 vs. LSS. si-PECAM-1: PECAM-1 siRNA; si-NC: negative control of PECAM-1 siRNA.

How LSS was sensed by cells and induced a series of intracellular changes? We then investigated whether PECAM-1 was involved in LSS induced inflammation. PECAM-1 siRNA was applied and verified (S3 Fig.). Compared with cells under LSS, PECAM-1 inhibition reduced PARP-1 expression and inflammatory response, including ICAM-1 protein expression, TNF-α and IL-1β secretion, and THP-1 cell adhesion (Fig. 6). The results suggested that PECAM-1 took part in the LSS induced inflammation response.

## Discussion

In the present study, we found that LSS induced the translocation of HMGB1 from the nucleus to cytoplasm and release to the supernatant. LSS could increase PECAM-1 and PARP-1 expression. PARP-1 was required for LSS-induced HMGB1 translocation and release and TLR4 expression. LSS could induce inflammatory response via PECAM-1/PARP-1/HMGB1 pathway.

Although atherosclerosis is associated with the classic risk factors of cardiovascular disease, it primarily affects large and medium arteries in a site-specifc manner. WSS has been proposed to regulate the site-specifc predilection of atherosclerosis.[5] As a type of WSS, LSS has a pro-atherosclerosis ability. LSS could increase oxidative stress, induce subendothelial accumulation of low-density lipoprotein,[31] decrease eNOS expression and NO production,[32] and induce expression of inflammatory molecules.[33] Wang has demonstrated that LSS increased inflammatory cytokines expression through activation of JNK and NF-κB.[29] In our experiments, we found that ICAM-1 expression was regulated by LSS in a time dependent manner, which verified that LSS could induce inflammation response during atherosclerosis. Then we investigate the mechanism by which LSS induced the inflammatory response. We focused on the nuclear protein HMGB1.

HMGB1 is a non-histone DNA-binding protein that acts as a cytokine when released in the extracellular milieu.[34] Extracellular HMGB1 can be considered to be a signal of tissue injury and a mediator of inflammation.[35] High levels of HMGB1 are found in inflammatory conditions such as sepsis.[36] We found that LSS could induce the translocation of HMGB1 from nucleus to cytoplasm and release to supernatant, accompanied with inflammatory response. HMGB1 inhibition by siRNA decreased LSS-induced inflammation response, such as ICAM-1 expression, TNF-α and IL-1β secretion, and monocytes adhesion. These results suggested a critical role of HMGB1 in LSS induced inflammation. Then we investigated how the LSS induced the translocation of HMGB1.

Poly(ADP)-ribose polymerase 1 (PARP-1) is a nuclear enzyme that transfers ADP-ribose moieties from NAD^+^ to itself and other proteins.[37] A role of PARP-1 in inflammation has been well described.[38] PARP-1 can regulate the expression of various key inflammatory genes including inducible nitric oxide synthase, intercellular adhesion molecule-1, and vascular cell adhesion molecule-1, all of which are regulated by NF-κB.[39,40] However, the role of PARP-1 in LSS induced inflammation has not been fully investigated. Our experiments found that inhibition of PARP-1 reduced LSS-induced inflammatory response. Then we investigated the underlying mechanisms. We found that PARP-1 inhibition could suppress the translocation of HMGB1 from nucleus to cytoplasm and release to supernatant. We also found that LSS increased TLR4 expression, while PARP-1 inhibition reduced it. Thus, HMGB1 could regulate inflammatory response through TLR4 in a PARP-1 dependent manner. However, the mechanism by which PARP-1 induced HMGB1 translocation and release needs further investigation.

How LSS was sensed by HUVECs and induced a variety of changes in cells? We then did further research on the question. There were various mechanosensors on cells, such as PECAM-1, integrin and caveolae. We chose PECAM-1 and inhibited it by siRNA. PECAM-1 inhibition reduced LSS-induced upregulation of PARP-1 and ICAM-1, which suggested that PECAM-1 took part in LSS induced inflammation.

In conclusion, we found that LSS increased PECAM-1 and PARP-1 expression. LSS could be sensed by PECAM-1 and induce inflammatory response via a PARP-1/HMGB1 dependent pathway. PARP-1 inhibition suppressed HMGB1 translocation and release and reduced TLR4 expression. Inhibition of PARP-1 might shed light on the treatment of HMGB1 involved inflammation during atherosclerosis.

## Supporting Information

S1 Fig HMGB1 siRNA reduced its mRNA and protein expression. HMGB1 was inhibited by siRNA, and then HMGB1 expression was assessed by RT-PCR and western blot analysis; **(A)** Quantification of HMGB1 mRNA expression. **(B, C)** The protein expression of HMGB1 was analyzed by western blot analysis. Values are expressed as mean ± S.D. from three separate experiments. *P<0.05 vs. USS; si-HMGB1: HMGB1 siRNA; si-NC: negative control of HMGB1 siRNA.
(TIF)

S2 Fig PARP-1 siRNA reduced its mRNA and protein expression. After PARP-1 was inhibited by siRNA, PARP-1 expression was determined by RT-PCR and western blot analysis. **(A)** Quantification of PARP-1 mRNA expression. **(B, C)** The protein expression of PARP-1 was analyzed by western blot analysis. Values are expressed as mean ± S.D. from three separate experiments. *P<0.05 vs. USS; si-PARP-1: PARP-1 siRNA; si-NC: negative control of PARP-1 siRNA.
(TIF)

S3 Fig PECAM-1 siRNA reduced its mRNA and protein expression. After PECAM-1 was inhibited by siRNA, PECAM-1 expression was determined by RT-PCR and western blot analysis. **(A)** Quantification of PECAM-1 mRNA expression. **(B, C)** The protein expression of PECAM-1 was analyzed by western blot analysis. Values are expressed as mean ± S.D. from three separate experiments. *P<0.05 vs. USS; si-PECAM-1: PECAM-1 siRNA; si-NC: negative control of PECAM-1 siRNA.
(TIF)
