# Supplementary figures and images for: Low Shear Stress Induced HMGB1 Translocation and Release via PECAM-1/PARP-1 Pathway to Induce Inflammation Response
Source: PLoS One. 2015 Mar 20;10(3):e0120586. doi: 10.1371/journal.pone.0120586 (PMC4368774; doi:10.1371/journal.pone.0120586)

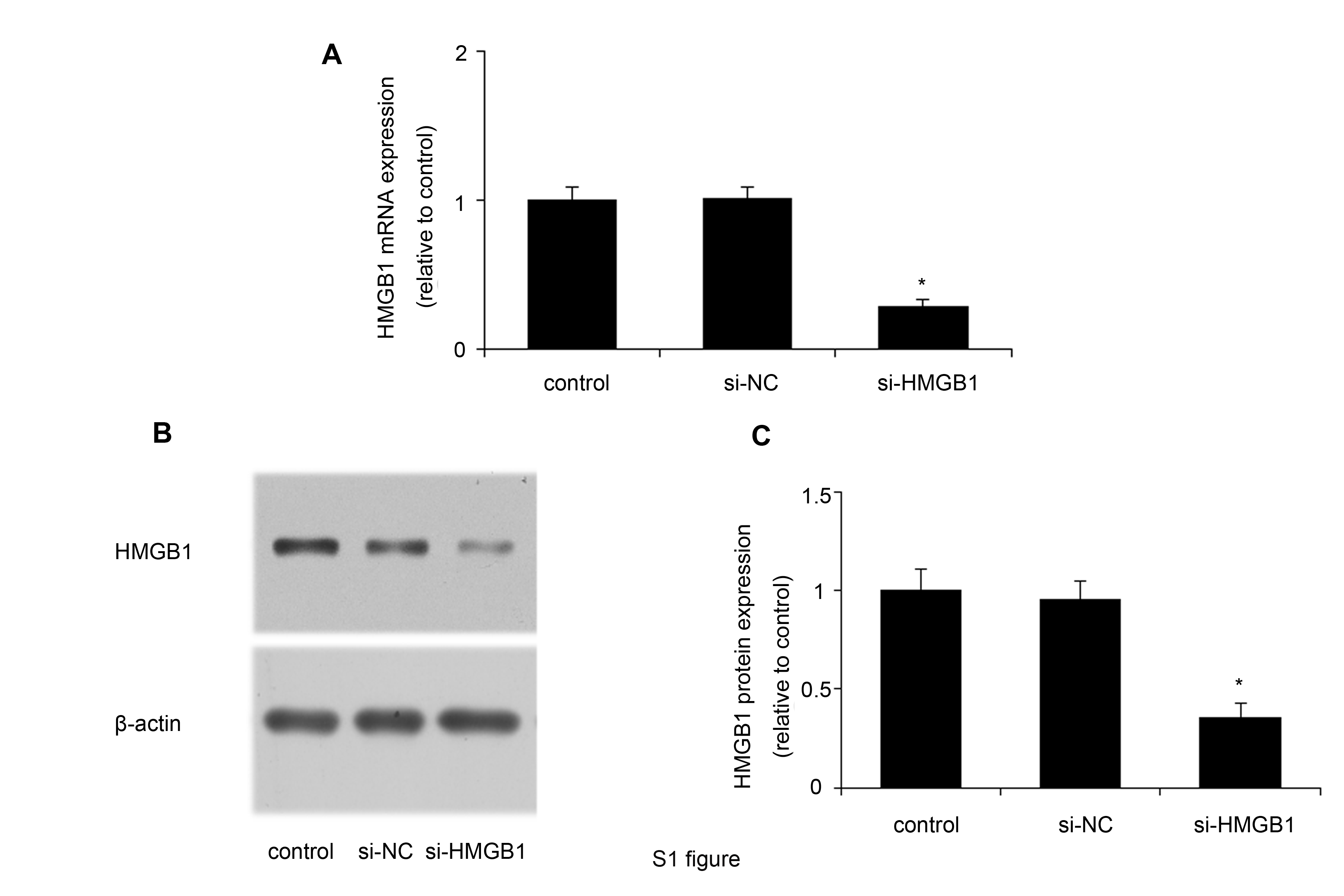

Supplement: S1 Fig — HMGB1 was inhibited by siRNA, and then HMGB1 expression was assessed by RT-PCR and western blot analysis; (A) Quantification of HMGB1 mRNA expression. (B, C) The protein expression of HMGB1 was analyzed by western blot analysis. Values are expressed as mean ± S.D. from three separate experiments. *P<0.05 vs. USS; si-HMGB1: HMGB1 siRNA; si-NC: negative control of HMGB1 siRNA. (TIF) [file pone.0120586.s001.tif]

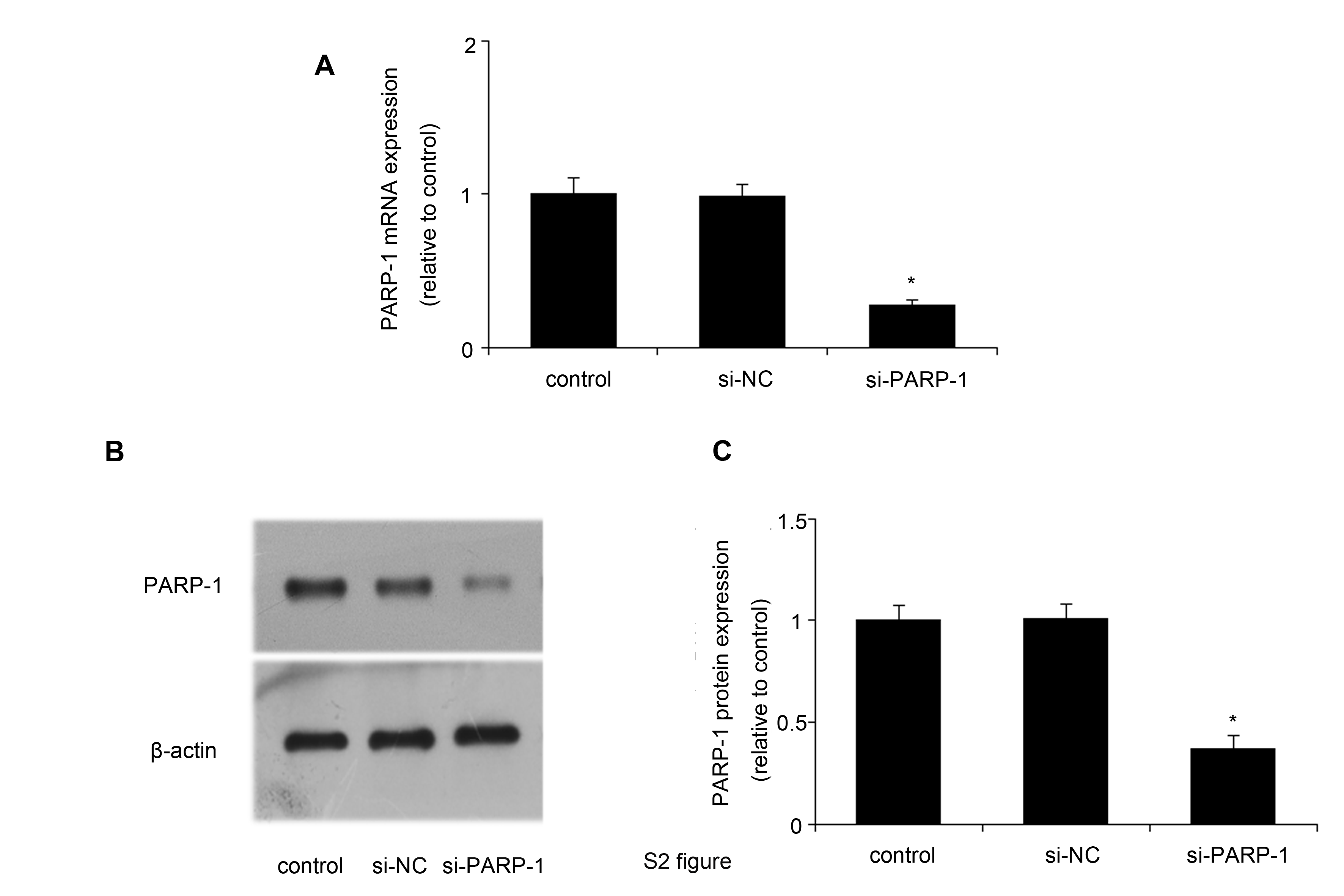

Supplement: S2 Fig — After PARP-1 was inhibited by siRNA, PARP-1 expression was determined by RT-PCR and western blot analysis. (A) Quantification of PARP-1 mRNA expression. (B, C) The protein expression of PARP-1 was analyzed by western blot analysis. Values are expressed as mean ± S.D. from three separate experiments. *P<0.05 vs. USS; si-PARP-1: PARP-1 siRNA; si-NC: negative control of PARP-1 siRNA. (TIF) [file pone.0120586.s002.tif]

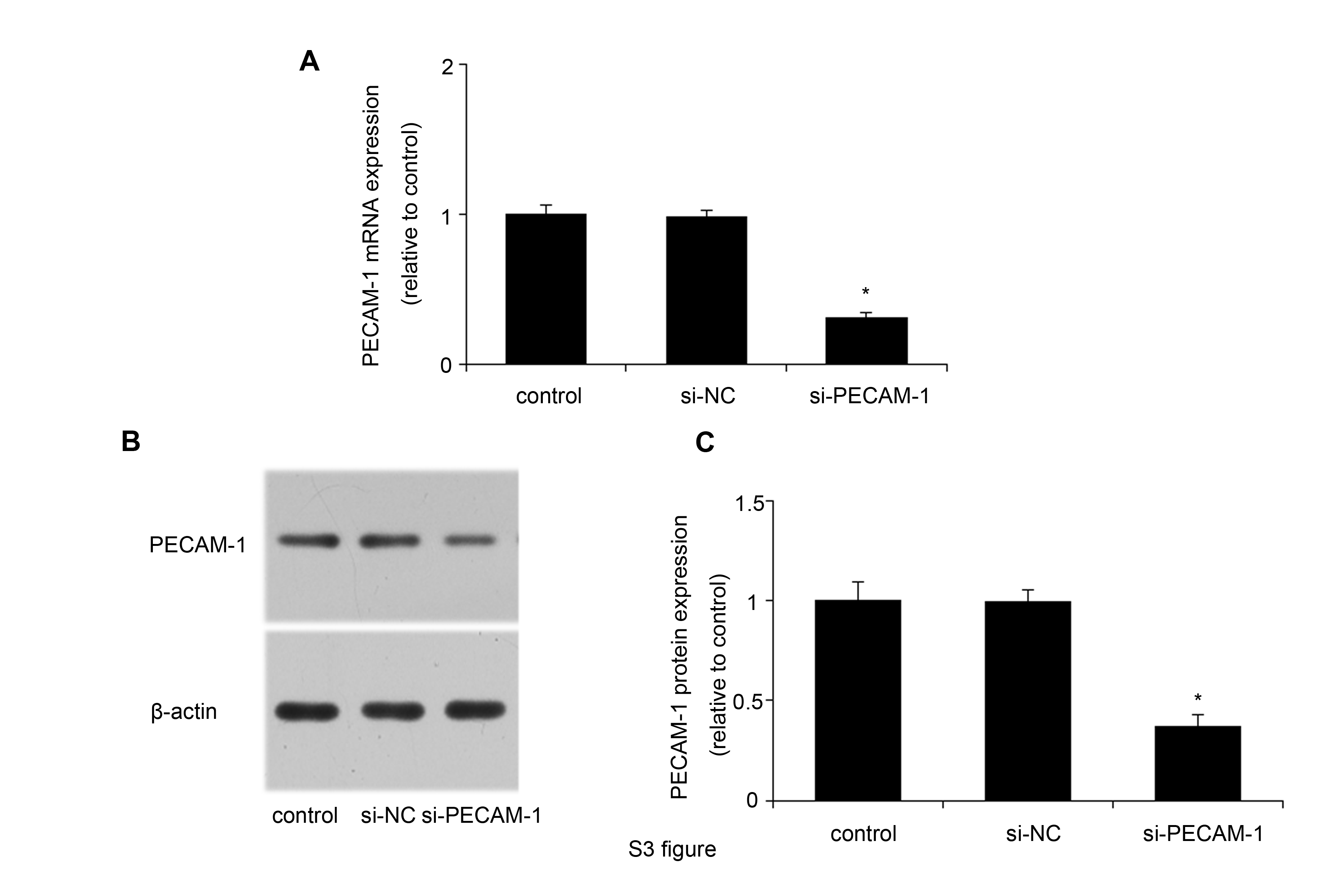

Supplement: S3 Fig — After PECAM-1 was inhibited by siRNA, PECAM-1 expression was determined by RT-PCR and western blot analysis. (A) Quantification of PECAM-1 mRNA expression. (B, C) The protein expression of PECAM-1 was analyzed by western blot analysis. Values are expressed as mean ± S.D. from three separate experiments. *P<0.05 vs. USS; si-PECAM-1: PECAM-1 siRNA; si-NC: negative control of PECAM-1 siRNA. (TIF) [file pone.0120586.s003.tif]
